# Through-Metal-Wall Power Delivery and Data Transmission for Enclosed Sensors: A Review

**DOI:** 10.3390/s151229870

**Published:** 2015-12-15

**Authors:** Ding-Xin Yang, Zheng Hu, Hong Zhao, Hai-Feng Hu, Yun-Zhe Sun, Bao-Jian Hou

**Affiliations:** Science and Technology on Integrated Logistics Support Laboratory, National University of Defense Technology, Changsha 410073, China; zhenghu@nudt.edu.cn (Z.H.); hongzhao@163.com (H.Z.); hhf_online@163.com (H.-F.H.); sunyunze@126.com (Y.-Z.S.); m18075163382@163.com (B.-J.H.)

**Keywords:** through metal wall, power delivery, data transmission, metallic enclosed sensors

## Abstract

The aim of this review was to assess the current viable technologies for wireless power delivery and data transmission through metal barriers. Using such technologies sensors enclosed in hermetical metal containers can be powered and communicate through exterior power sources without penetration of the metal wall for wire feed-throughs. In this review, we first discuss the significant and essential requirements for through-metal-wall power delivery and data transmission and then we: (1) describe three electromagnetic coupling based techniques reported in the literature, which include inductive coupling, capacitive coupling, and magnetic resonance coupling; (2) present a detailed review of wireless ultrasonic through-metal-wall power delivery and/or data transmission methods; (3) compare various ultrasonic through-metal-wall systems in modeling, transducer configuration and communication mode with sensors; (4) summarize the characteristics of electromagnetic-based and ultrasound-based systems, evaluate the challenges and development trends. We conclude that electromagnetic coupling methods are suitable for through thin non-ferromagnetic metal wall power delivery and data transmission at a relatively low data rate; piezoelectric transducer-based ultrasonic systems are particularly advantageous in achieving high power transfer efficiency and high data rates; the combination of more than one single technique may provide a more practical and reliable solution for long term operation.

## 1. Introduction

In condition monitoring and wireless sensing applications, it is common to encounter the problem of how to power and communicate with sensors enclosed in sealed metal containers, vacuum/pressure vessels or located in a position isolated from the operator by metal walls (e.g., vessel hulls and bulkheads, aircraft and spacecraft fuselages, vehicle armor). Of course sensors can be powered using batteries, but nevertheless, on some occasions, batteries are not suitable as power sources due to the nature of the installation environment, volume or other constraints. Furthermore, when batteries are used, the operation complexity and cost of replacing batteries in these metal enclosures become problematic.

Traditionally, metal wall penetrations are used to feed through wires. However, when using wires to transfer power and data through a metallic structure, there are many practical design problems needed to be taken into account such as the probability of leakage of toxic chemicals, loss of pressure or vacuum, as well as difficulties in handling thermal and electrical insulation. Moreover, feeding wires through a metallic wall of a structure reduces the strength and integrity of the structure and places the structure at high risk of cracking due to stress fatigue. Meanwhile it will also increase the total lifetime maintenance costs.

According to a news release from British AErospace (BAE) Systems ([1], a single submarine requires up to 300 penetrations of the hull to allow for the passage of cables transmitting power and carrying data for monitoring sensors and other devices. These penetrations are expensive to fit and require additional strengthening of the hull to counteract structural stress fatigue, which substantially increases through-life maintenance costs.

In the modern aeronautic and aerospace areas, there is also a strong need for reliable sensors which can be operated and powered wirelessly, especially for maintenance and monitoring purposes. During the Mars Sample Return Mission conducted by NASA, the sealed sample container will use wireless sensors to sense pressure leaks and to avoid potential contamination, where power and data need to be transmitted through the metal container wall without penetration [2]. For the wireless sensing and health monitoring of aircraft, a solution allowing successful transmission of power and data through metal walls for sensors embedded in conductive materials without any physical penetration is also in urgent demand [3].

According to the published literature reports, there have been several solutions for through-metal-wall power delivery and/or data transmission without intrusive procedures like drilling holes in the metal walls. Among these solutions, one category of solutions is called electromagnetic coupling-based methods, which involve inductive coupling [4,5,6,7,8], capacitive coupling [9,10,11] and magnetic resonance coupling [12]. Some amount of power and data transmission through metal walls can be achievable using electromagnetic-based solutions. However, due to the strong Faraday shielding effect or skin effect presented by ferromagnetic metallic barriers or thick non-ferromagnetic metallic barriers, their effectiveness is inhibited, making them highly inefficient and impractical. In the review, we will provide a brief description of these methods.

Another category of solutions is called acoustic/ultrasound-based methods, which are more efficient and have been receiving considerable attention in recent years since ultrasonic waves can propagate easily through various kinds of metals and solids.

In this review, we first describe the significance and requirement of through-metal-wall power delivery and data transmission for monitoring sensors in metallic enclosures. Then we: (1) describe three electromagnetic coupling-based techniques reported in the literature, which include inductive coupling, capacitive coupling, and magnetic resonance coupling; (2) present a detailed review of wireless ultrasonic through-metal-wall power delivery and/or data transmission methods and systems; (3) compare various ultrasonic through-metal-wall systems in acoustic-electric channel modeling, transducer configuration and communication mode with sensors; (4) summarize the characteristics of electromagnetic-based and ultrasound-based systems. Finally, we provide our conclusions and an overview of the challenges and development trends in through-metal-wall power delivery and data transmission.

## 2. Methods for Through-Metal-Wall Power Delivery and Data Transmission Based on Electromagnetic Coupling

To transmit power and data based on electromagnetic coupling is not a new concept. However, wireless transmission of power and data through metallic barriers is quite different. Due to the shielding effect or skin effect in metal, electromagnetic waves cannot effectively pass through metal, and especially ferrous metal barriers. However, there have still been three kinds of electromagnetic coupling-based methods achieving this target according to literature publications: inductive coupling [4,5,6,7,8], capacitive coupling [9,10,11] and magnetic resonance coupling [12]. We will describe each of these methods respectively in the following subsections.

### 2.1. Power Delivery and/or Data Transmission Based on Inductive Coupling

Inductive coupling method can be used to transfer power and/or data through thin metal barriers. The technology of inductive power transfer (IPT) is probably the most popular wireless power transfer approach nowadays. It works by magnetic field coupling using a pair of coils.

#### 2.1.1. Inductive Power Transfer Technology through Metal Walls

The principle of IPT is similar to the principle of transformers. In a transformer, an alternative magnetic field is produced by a current flowing in the primary coil. An induced current is generated in the proximate secondary coil by the principle of electromagnetic induction. The primary and secondary coils are closely coupled using a common silicon steel plate or iron core in a transformer, whereas an IPT system comprises detached primary and secondary coil parts.

Though IPT has achieved great success in theoretical development and industrial applications for wireless power delivery in certain areas [13,14,15], it is rarely used for power transfer through a conductive metal because generally the metallic medium restricts the power transfer due to losses causing by the shielding effects. However, for low energy applications, several publications have shown that IPT can be used to transfer an acceptable amount of power through thin metal walls [4,5,6,7,8].

**Figure 1:**
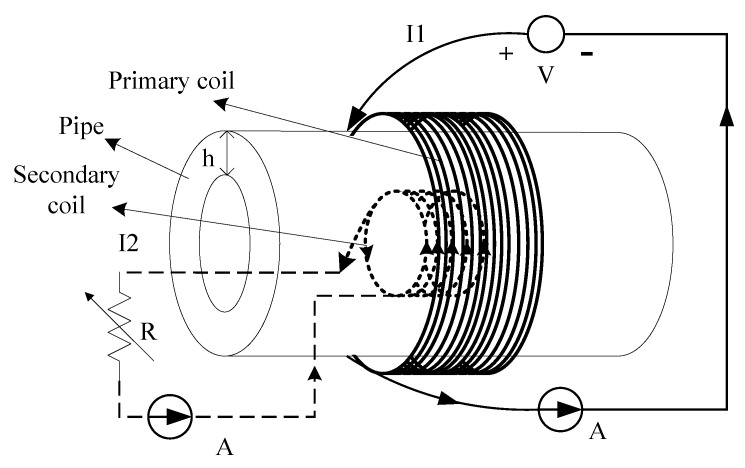
Diagram for power transfer through a metal pipe using the IPT method, redrawn from Imoru *et al.* [4].

Imoru *et al.* [4] studied the feasibility of power transfer through metal pipes using an analytical model governing the mechanism of IPT. The analytical model results were also checked experimentally. In the experiment validation, two different steel pipes are used as testing objects between the primary and secondary coils. The first is stainless steel, the second is a ferrous steel material and both of them have outer and inner diameters of about 220 mm and 200 mm. The experimental setup is shown in Figure 1. The primary coil (about 320 turns with diameter of 240 mm) outside the pipe is connected to the power supply. The secondary coil (about 220 turns with diameter of 185 mm) inside the metal pipe is connected to the load. Inductive power is transferred from the primary to the secondary coil through a thin metal wall. It is found that the maximum efficiency of the system for the stainless steel pipe lies between the frequencies of 40 Hz to 100 Hz with an output load of 5.55 Ω. A maximum efficiency of over 22% is achieved at a frequency of 100 Hz. To the contrary, there is no detectable current when a ferrous steel pipe of high permeability is used as the barrier between the primary and secondary coils.

In [5,6], Graham *et al.* analyzed inductive power transfer for through metal applications by means of equivalent circuit modeling, finite element simulation and experimentation. The power transfer efficiency variations with frequency through a 20 mm thick, 130 mm diameter stainless steel disc of a relative permeability of μ*_r_* = 1.1 were obtained. The results showed that an efficiency exceeding 5% can be achievable with an operating frequency of 500 Hz. However, the authors also point out that when the relative permeability of the metal material is increased to μ*_r_* = 10, the peak achievable efficiency is only 0.2%, which starts to make IPT impractical. Materials of high permeability are not suitable to transfer any power at any practical thickness using the IPT method. which coincides with the conclusions reached in [4].

#### 2.1.2. Inductive Power Transfer and Through-metal-wall Data Transmission

Besides power delivery, the inductive coupling method can also be used for simultaneous data transmission through metal walls. For sensors enclosed in a sealed metal structure under extreme conditions such as high pressure, small volume and required mechanical stability, simultaneous through-metal-wall power transfer and data transmission is of great importance for sensors without penetration. Zangl *et al.* [7,8] investigated the feasibility of power delivery and data transmission through a metal pipe and a metal container’s walls by means of finite element analysis and laboratory experiments based on the previously discussed IPT technique. The simulation demonstrated that super low frequency (30 Hz–300 Hz) continuous carriers are suitable for power delivery, whereas, for data co mmunication with the sensor, higher frequencies in the low frequency range (30 kHz–300 kHz) domain can be used.

**Figure 2:**
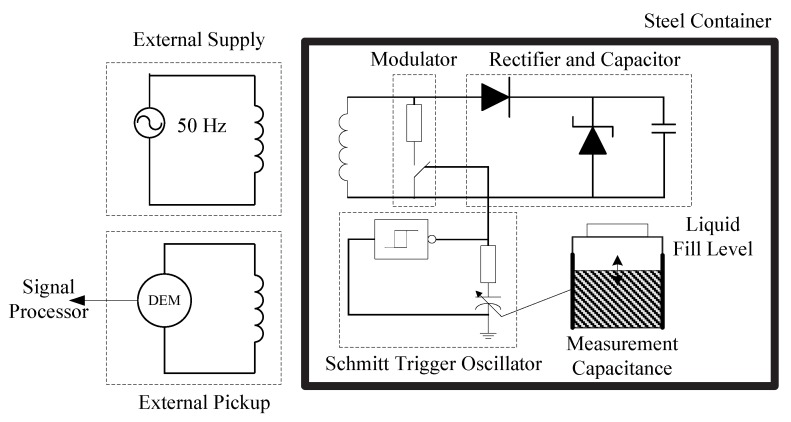
Diagram of power delivery and data transmission for an fill level capacitive sensor electronics enclosed in a metal container, redrawn from Zangl *et al.* [8].

For the metal container situation, the external coil uses a ferromagnetic core to increase the magnetic field. The load modulation signal is observed with a separate pick-up coil for the purpose of simplified separation from the carrier signal. A fill-level-dependent capacitor is chosen for the metal container setup in the experiment. A block diagram of through-metal-wall power and data transmission for a liquid fill level capacitive sensor enclosed in a metal container is shown in Figure 2.

In Figure 2, the external coil is directly powered from a variable transformer using a continuous wave carrier signal at 50 Hz. The sensor electronics inside the tank consist of a rectifier and a capacitor as energy storage, a Schmitt trigger oscillator, a metal oxide semiconductor field-effect transistor (MOSFET) switch for load modulation, and a variable resistor for frequency adjustment. The liquid fill level sensing capacitor is connected to the Schmitt trigger oscillator. The capacitance variation due to the changes in the liquid fill level is reflected in the oscillation frequency variation of the oscillator, which modulates the carrier signal. For the easy separation between the carrier and the modulated signal, a separate pick-up coil outside the tank is used to receive the response from the sensor. The load modulation frequency is chosen to be greatly higher than the carrier frequency in the range of 60 kHz–180 kHz.

The experiment results have shown that at least 30 μW of power can be transferred through the 0.5 mm thick tin tank wall to power the fill level measurement capacitive sensor circuit using a continuous wave carrier signal at 50 Hz. The load modulation signals can be successfully picked up outside the container. It is estimated that a data rate of 20 kbps can be achieved between the sensor and the external unit. However no through-metal-wall power transfer efficiency information was presented in the paper.

In [6] Graham suggested inductive communication can be used for applications requiring low data rates with power transfer through metal barriers to simultaneously power the sensor electronics. The experiments show that a data rate in excess of 100 bps through a 20 mm test stainless steel plate can be achieved. Furthermore, it is asserted that the communication data rate will never exceed a few kbps due to the inherent bandwidth limitations.

### 2.2. Power Delivery and/or Data Transmission Base on Capacitive Coupling

Power delivery through metal based on capacitive coupling is usually termed Capacitive Power Transfer (CPT), or Capacitively Coupled Power Transfer (CCPT). Unlike IPT using magnetic field coupling, CCPT provides an alternative way of wireless power transfer based on electric field coupling.

**Figure 3:**
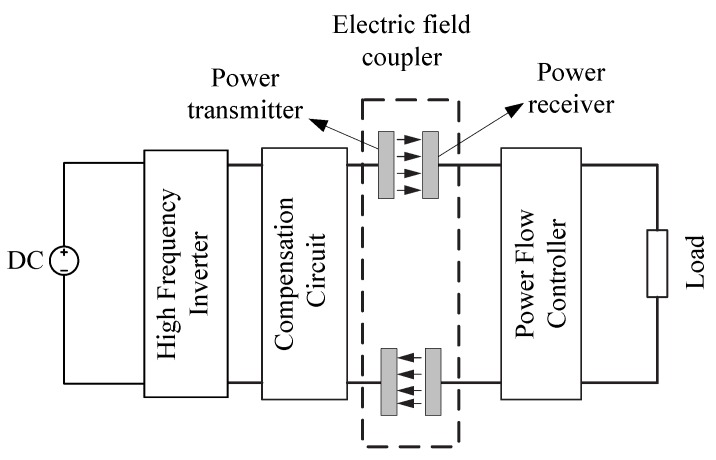
The structure of a typical CCPT system, redrawn from Huang *et al.* [11].

Figure 3 shows the typical structure of a CCPT system, which consists of three key components, namely a primary power transmitter, electric field coupler, and power receiver which regulate the collected power and providing power supply for the load.

The power transmitter acts as a high frequency voltage source. High frequency AC voltage is supplied to two primary metal plates. When two secondary plates are placed close to the two primary plates, electric fields are formed and there is a displacement current between them. As a result, power can be transferred to supply the load without direct electrical contact.

The capacitive electric field coupling part is the key element of the CPPT system, acting as the power transfer channel. Its structure and coupling condition affect the power transfer capacity directly. The electric field coupler functions as two capacitors connected in series during the operation of CCPT. According to different applications, the capacitive coupling structure can be of various configurations like rectangular, cylindrical, disk or matrix structures [11].

When there is a metal barrier between a pair of coupling plates, the electric field coupling can be regarded as a series connection of two capacitors and power can still pass through the intermediate metal barrier. This means that CCPT has the ability to deliver power through metal walls. A demonstrated CCPT system has been developed to transfer power from a DC power supply to a lamp and a calculator with metal barriers and without metal barriers, respectively [9]. Although there is no information about the amount of power transferred through the metal barrier and the power transfer efficiency, the previous system has demonstrated that wireless power transfer through a metal barrier to multiple loads using CCPT technology is feasible. However, according to the principle of the CCPT method, the metal barriers in two pairs of coupling plate should be separated from each other, so is not feasible to transfer power through a sealed metallic structure to inside sensors using the CCPT method, and up to now there are no reports about through-metal-wall data transmission using the capacitive coupling method.

### 2.3. Power Delivery through Metal Based on Magnetic Resonance Coupling

**Figure 4:**
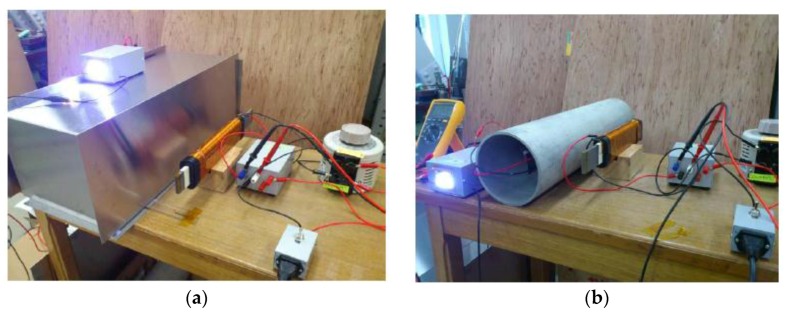
Experimental demonstration setups (**a**) power transfer through a space enclosed by stainless steel metal walls of 1 mm thick; (**b**) power delivery through a metal pipe of 5 mm thick, from Yamakawa *et al.* [12] (Open Access).

Power transfer based on magnetic resonance coupling was first proposed by MIT researchers [16]. They developed a system calling “Witricity”, which can wirelessly transfer 60 watts with an efficiency of 40% over a distance of 2 meters in air. It greatly enlarges the efficiency of power transfer over large distances and has drawn attention from all over the world [17,18,19,20]. However using magnetic resonance coupling for through-metal-wall power transfer is very rare. In 2014, Yamakawa *et al.* [12] at the University of Tokyo reported their work on wireless power transmission into a space enclosed by metal walls using magnetic resonance coupling. In the paper, a wireless power transmission system using magnetic resonance coupling was proposed and demonstrated for supplying power to electrical sensors or devices working in a space enclosed by metal walls. Differing from the resonance frequency usually chosen in the kHz-MHz range to obtain a high quality factor of several hundred in conventional magnetic resonance, the demonstrated system is driven at a low resonance frequency of 50 Hz, which is deliberately selected to avoid eddy current losses in the enclosed metals. After theoretically analyzing the equivalent circuits and the transmission efficiency based on magnetic coupling, two kinds of through-metal-wall experiments are carried out. The results have shown that 3 W of electric power can be supplied to LEDs through a stainless steel wall 1 mm thick with an efficiency of approximately 40% over a transmission distance of 12 cm. In another experiment, 1.2 W of power was transmitted to LEDs through a 5 mm thick metal pipe with an efficiency of 10%. The experimental setups are shown in Figure 4. Though the IPT method uses the same frequency [7,8], according to the authors, the power transmission efficiency is improved markedly using the magnetic resonance coupling method.

In another work, for the purpose of charging batteries operating in structures that humans cannot enter, such as areas contaminated by radioactivity, Ishida *et al.* [21] developed a method for transmitting electrical power through concrete walls based on magnetic resonance coupling. Electric power (165 W) was transmitted over a distance of 100 mm with an efficiency of about 67% through a concrete plate containing a steel frame. However the barrier is not a metal barrier in the full sense.

Up to now there are also no literature reports on through-metal-wall data transmission using the magnetic resonance coupling method, although in theory it can be competent for this task. In a patent titled “Magnetic communication through metal barriers”, a wireless data transmission system through non-ferrous metal barrier based on magnetic field coupling was described [22]. The transmitter and receiver coils wound around an iron core attached close to the opposite sides of the metal barrier, respectively. The operating frequency range is in kilohertz range. The patent claims it can transmit power through a metal barrier for powering the sensors, yet there is no experimental evidence to support this.

It can be summarized that the previously discussed electromagnetic principle-based through-metal-wall power and/or data transmission systems can only apply to non-ferromagnetic metals and thin metal walls due to the Faraday shielding effect. Therefore, the electromagnetic principle is only suitable for power transfer and data transmission at a relative low data rate through thin metal walls of low permeability such as stainless steel, aluminum, titanium, *etc*. Compared with electromagnetic waves, acoustic waves, especially ultrasonic waves, have excellent propagation characteristics in both non-ferromagnetic and ferromagnetic materials, making it an attractive concept using ultrasound for power delivery and data transmission through metal walls.

## 3. Ultrasonic Through-Metal-Wall Power Delivery and Data Transmission Technology

Acoustic waves, especially ultrasonic waves, have widely been used in fields such as underwater acoustic communications [23,24], industrial detection and health monitoring of structures [25]. Wireless ultrasonic power transfer has been used to power biomedical implants through human tissues [26,27,28,29], so this should be a favorable solution for power delivery and data transmission through metal walls by means of ultrasound. However, this field is less well developed than areas such as underwater acoustic communications. Only in the past several decades, methods of ultrasonic through-metal-wall power delivery and data transmission are getting more attention and have been investigated by a number of research groups.

### 3.1. Initial Development of Ultrasound Through-Metal-Wall Power Delivery and Data Transmission

In a traditional ultrasonic inspection system, an acoustic wave is injected into a metal by means of a piezoelectric transducer (PZT) coupled to the surface, and the reflections of the acoustic waves are analyzed. This sort of system has demonstrated the ability to readily transmit energy through a metal wall, and it should be reasonable to improve such a system for through-metal-wall power delivery and/or data transmission.

The first idea of transferring power and sensor data through metal walls by means of ultrasonic waves was presented in a patent by Connor *et al.* in 1997 [30]. The proposed system was comprised of two coaxially aligned PZTs attached on opposite sides of a solid wall, a signal generator, and all the supporting circuitry that performs analog-to-digital (A/D) conversion, modulation of the sensor data and power harvesting on the inside, and demodulation of the digitized data on the outside. The signal generator applies a continuous wave (CW) electrical signal to the transmit transducer to create ultrasonic vibrations. The ultrasonic waves produced by the outside transmitting transducer would propagate through the wall and cause ultrasonic vibrations in the inside receiving transducer and the vibrations are then converted back to electrical signals. Thus, an electrical signal could be applied to one transducer, converted into acoustic energy that propagates through the metallic barrier, then converted back into an electrical signal by a second transducer.

Moreover, the electrical signal generated by the inside transducer can be harvested using power harvesting circuits to provide power for the inside sensor circuitry. The author suggests a novel method for sending sensor data from inside to outside by modulating the impedance of the inside transducer. A MOSFET, acting as an electronic switch, is added across the inside transducer’s terminal. The electrical impedance across the terminals of the transducer, which is related to its acoustic impedance, would be changed by opening and closing this MOSFET switch. The change of the inside transducer’s acoustic impedance would induce a variation of the acoustic energy reflected back to the outside transducer, thus meaning sensor data can be sent back from the inside electronics to the outside on the same signal applied from the outside. In this way, only two transducers are needed for simultaneous power delivery and data transmission. However, the author does not provide information about the power transfer amount and transfer efficiency through the wall to the other and the report also fails to provide the data rate from one side of the wall to the other. Nevertheless, the patent illustrates the fundamentals of simultaneously transmitting power and data through the metallic barriers.

Another two patents titled “Ultrasonic data communication system” and “Ultrasonic power communication system” were assigned to Welle [31,32]. In these patents, the proposed system shares many similarities with the previously presented system in [30]. The system utilizes a pair of PZTs which are coupled directly to opposite sides of a metal wall. The system contains both electronics on both sides of the metal wall for signal modulation and recovery. The collection of electronics located on isolated, side of the metal barrier is denoted the “embedded sensory & actuating unit”, and the collection of electronics located on the accessible, *i.e.*, non-isolated, side of the metal barrier is denoted the “external controller”. Reference [31] focuses mainly on the data transmission aspect with the sensor isolated by the metal barrier, while [32], focuses primarily on the delivery of power, not data. The embedded sensory and actuating unit in this system rectifies received electrical energy from the CW signal and stores it in a rechargeable battery through power conditioning circuits. A similar system was also presented in a patent titled “Remote Energy Supply Process and System for an Electronic Information Carrier” by Rein in 2003 [33]. However, just as in [30], there is no measurement or performance information provided for these patented system, and it also appears that there are no acoustic-electric models of these systems.

**Figure 5:**
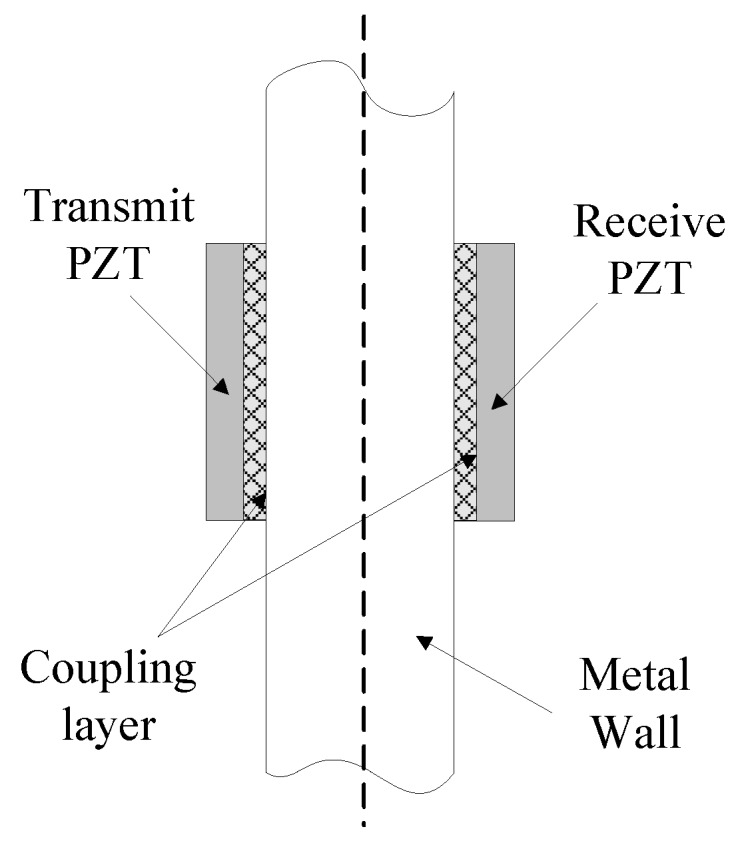
Diagram of PT channel formed by a pair of piezoelectric transducers.

Henceforward, the ultrasonic piezoelectric transducers are utilized by many research groups as a mean of power and data transfer through metal walls. A pair of PZTs coaxially coupled directly to opposite sides of the metal wall form an acoustic-electric channel which is often called a piezoelectric transformer (PT). The diagram of the PT channel is shown in Figure 5. Such an acoustic-electric channel is the most common ultrasonic system configuration under study because PZTs can be kept small and very efficient with careful material selection.

More in-depth and detailed researches have been carried out and many ultrasonic PZT-based systems have been proposed. Some researchers mainly focus on power delivery alone, while some focus on data transmission alone, and some others focus on simultaneous power and data transmission.

### 3.2. Ultrasonic Through-Metal-Wall Power Delivery

In 2003, Hu *et al.* [34] showed that transmission of electric energy into a sealed armored space can be achieved through thickness-stretch vibrations of the metal wall together with two PZTs coupled directly opposite each other on the metal wall, one as the transmitting transducer and the other as the receiving transducer. A sinusoidal voltage is applied across the transmitting transducer at a known frequency generating an ultrasonic wave that travel through the wall into the receiving transducer where the stress wave generates a sinusoidal voltage. The output voltage, the output power, and the efficiency of this system are solved using the wave propagation equation and linear equations of piezoelectricity theoretically. In [35] the power delivery channel consisting of an elastic plate with finite piezoelectric patches on both sides of the plate is studied. It is shown that when thickness-twist mode is used, the structure shows energy trapping with which the vibration can be confined to the transducer region.

These previous work were followed by several papers focusing on physical and mathematical modeling of piezoelectric transducer-based power delivery through metal walls of different geometric shapes with different vibration modes. In [36,37,38] the modeling of three-layered cylindrical shell PT configurations are presented, where a pair of cylindrical PZTs are aligned concentrically on the inside and outside of a cylinder of elastic material. These transducers are acoustically coupled to the elastic layer’s inner and outer surfaces operation in the thickness-twist mode of vibrations [36], thickness-shear mode of vibrations [37], and thickness-stretch mode of vibrations [38], respectively. Efficient power harvesting methods and circuits are also considered [39,40,41] when modeling the power transfer capabilities of some of these channels.

The work reported in these papers highlights the important points that a coupled PT system’s power transfer capabilities are sensitive to the driving frequency, the mechanical properties of the piezoelectric and barrier materials used, and the electrical loading presented to the transducers. Therefore, careful design is needed for optimal power delivery performance. However, it should be pointed out that only numerical results are presented in these literatures. No practical systems are developed, so no physical measurements are performed to validate the accuracy of the models, or to support the conclusions drawn from these studies.

Since 2005, a research group at U.S. Jet Propulsion Laboratory has studied what they call “wireless acoustic-electric feedthroughs” for power delivery through metal walls to sensors enclosed in sealed metal tank for the requirements of NASA applications [2,42,43,44,45,46]. Sherrit *et al.* [2] proposed a network equivalent circuit model to study the power transmission performance of the same sandwiched plate PT power delivery channel as provided in [34]. The equivalent circuit model method is superior to wave propagation equation and piezoelectric theory-based methods in modeling the acoustic-electric channel because the equivalent circuit can be easily modified to account for additional acoustic elements such as the insulation layer for the transducer, coupling layer between the transducer and the metal wall. The circuit model can also be connected to other circuits or networks such as driving source circuit, impedance match circuit, and load circuits. Identical results were obtained by the equivalent circuit model when using the same material parameters as in [34]. The model was also extended in the paper to include dielectric, piezoelectric and mechanical losses in the walls.

Subsequently, several papers were published by the same group. In [42] the influences of different coupling schemes between the transducer and the plate on the efficiency of power delivery through a 2.5 mm thick titanium plate were studied in detail. The coupling modes include bolted using a backing structure, clamped with grease couplant covering the contacting surface, and attached using a conductive epoxy. The mechanical clamp coupling mode is shown to get the highest measured efficiency of 53%, but this technique is impractical since it requires a clamp spanning the titanium wall. Using a conductive epoxy was found to give a reasonably high transfer efficiency of 40% by eliminating that impracticality. In [43,44] finite element models (FEMs) were developed to calculate the power loss due to energy carried away by the traveling Lamb waves. The authors have shown clearly that Lamb wave loss should be an important factor when designing a sandwiched plate power delivery channel, especially for thick metal walls.

The subject of high power delivery through thin metal plates was studied in [45,46]. The experimental results showed that 100 W of power was transferred through a 3.4 mm thick titanium plate using epoxy coupling and a pair of 38 mm diameter disc PZTs [45]. Pre-stressed piezoelectric stack transducers were used for ultra-high power delivery through a 5 mm thick titanium plate. The experimental results have shown that 1083 W of power can be transmitted through the plate at an efficiency of 84% with a driving frequency of 24.5 kHz by means of four 50.8 mm diameter disc PZTs stacked on each side of the structure [46]. The diameter, thickness and the number of PZTs of the stack can be adjusted to meet the power and impedance match requirements. FEM simulations were also presented in the paper to illustrate that Lamb wave loss can be greatly reduced by attaching a ring reflector to both sides of the titanium plate.

Another group at the Australian Defense Science and Technology Organization published several technical reports and papers on the modeling and development of systems for power delivery and data transmission through aluminum metal walls based on ultrasound [47,48,49,50,51]. Moss *et al.* [47,48] investigated an ultrasonic acoustic electric feedthrough (AEF) to transfer power through a metal plate for *in situ* structural health monitoring sensors embedded within aircraft and other high value engineering assets. An equivalent circuit model of the AEF system based on LTSpice^®^ circuit software simulation was presented to describe the electro-mechanical behavior of the power transfer channel. The system was then validated using experimental data. The results have shown that by using a pair of 38 mm disc piezoelectric transducers with thickness-mode resonance at 1 MHz, as much as 300 mW of power can be transmitted through the aluminum plate with thicknesses in the range of 1.6 mm–5 mm at an efficiency of 30%. Furthermore, an upgraded system incorporating both power delivery and data transmission through aluminum metal walls was developed in [49,50]. The system is able to transmit 420 mW of power through 1.6 mm thick aluminum plate to the electronic components at an efficiency of 42%, as well as transfer data at rates as high as 115 kbps. In [51] a detachable acoustic electric feedthrough to transfer power and data across a metal (or composite) plate was developed and characterized. Two axially aligned piezoelectric-magnetic structures were mounted on opposite sides of a 1.6 mm thick aluminum plate, and aligned by magnetic force automatically using NdFeB magnets. The system was experimentally validated that 340 mW of power can be transferred at an efficiency of 34%. Simulation even demonstrated that simultaneously transferring data at a rate of 115 kbps is feasible.

### 3.3. Ultrasonic Through-Metal-Wall Data Transmission

Through-metal-wall power delivery has received much attention. Meanwhile, some research groups have focused mainly on through-metal-wall data communication with sensors enclosed in sealed containers.

In 2000 Hobart *et al.* [52] at Woods Hole Oceanographic Institution developed a device called the “HullCom” acoustic modem unit (AMU) for transmitting data using a ship’s hull or frame as the acoustic path, which is the earliest report concerning through metal data transmission. The concept of using two separate pairs of piezoelectric transducers to form forward and reverse transmission channels for bidirectional data communication through a ship hull was proposed by Payton in a patent titled “System for acoustically passing electrical signals through a hull” [53]. However, it is unclear whether or not a physical system was ever built to test and validate the concept. A similar system was also proposed for data transmission through a submarine hull using high frequency ultrasonic signals in a patent titled “Data transfer” [54]. Though sensors were not mentioned in the patent, the proposed method is suitable for through-metal-wall data transmission for sensors isolated by metal walls.

In 2006, a research group at Rensselaer Polytechnic Institute started to research wireless ultrasonic data transmission through metal walls. Murphy [55] in his master’s thesis investigated the ultrasonic methods for low rate environmental sensor monitoring (*i.e.*, temperature, pressure, *etc.*) inside a sealed pressure vessel. A “single-hop” unidirectional data transmission system for unidirectional data transmission, a “double-hop” full-duplex system with separate forward and reverse transmission channels, and a “reflected-power” configuration where bidirectional data transmission occurs in the same acoustic-electric channel have been developed and demonstrated on a 148 mm thick steel wall. The achievable low data rates are respectively 500 bps, 5 kbps, and 300 bps for these three configurations. In [56] a further “hybrid” system approach which merges the ideas behind the “double-hop” and “reflected-power” system was presented. It is able to communicate with a temperature sensor at a rate of 500 bps through a 152.4 mm thick steel wall.

A research group at Drexel University is dedicated to studying high-data-rate ultrasonic data transmission through metal walls using sandwiched plate PT configurations [57,58,59,60,61,62]. Though the target of their work of developing an ultrasonic system is not for enclosed sensor data communications but for wirelessly repeating data across the bulkheads separating watertight compartments of naval vessels, these two aspects essentially coincide with each other. They investigated a wide variety of channel equalization techniques and digital communication schemes to improve the transmission data rate from 50 kbps to 30 Mbps, including echo-cancellation based pre-distortion filter to suppress strong channel reverberation [57], pulse amplitude modulation (PAM) scheme [58], and an orthogonal frequency-division multiplexing (OFDM) digital modulation scheme [59,60,61,62].

Hosman *et al.* [63,64] at the University of Oklahoma have developed an economic (multitone frequency-shift keying) MFSK communication system based on ultrasonic signals transmitting through steel shipping container corner posts. This system can fulfill the requirement of transmitting information to/from device located inside a closed metal shipping container in transit. The ability to reliably encode and transmit 18 bits of data per modulated MFSK symbol was demonstrated. It can transmit data at a rate of 360 bps, which is applicable to low data rate requirement applications.

### 3.4. Simultaneous Through-Metal-Wall Power Delivery and Data Transmission Based on Ultrasonic

**Figure 6:**
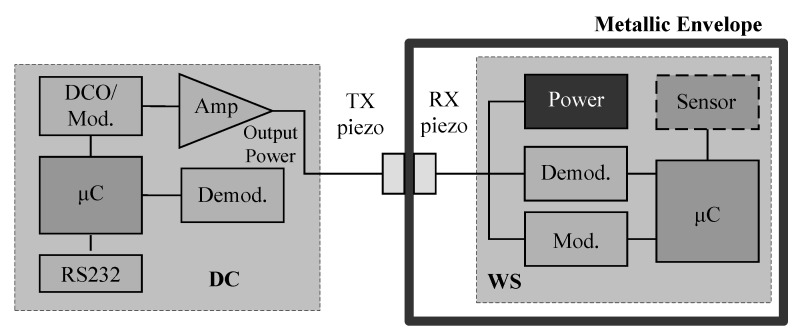
Ultrasonic transmission system block diagram, redrawn from Kluge *et al.* [3].

In the meantime several research groups have focused on the study of simultaneous power delivery and data transmission through metal barriers based on ultrasonic waves. Since 2007, some researchers at EADS Innovation Works, University of Paderborn, and Saarland University [3,65,66] have collaboratively studied remote ultrasonic power delivery and data transmission for sensors inside sealed metal containers, like conductive envelopes, hydraulic accumulators, oxygen bottles, *etc.* for aeronautic and aerospace applications. In [3,65] a sandwiched PT channel was formed on a 7 mm thick aluminum barrier using PZTs with thickness-mode resonances near 1 MHz. A wireless sensing system was developed. The block diagram of the system is illustrated in Figure 6.

It consists of two parts: the data concentrator (DC), which is located outside the metal envelope and the wireless sensor (WS), which is isolated inside the envelope. The data concentrator generates the carrier frequency to drive the TX piezo in resonance and allows transferring energy from the transmitter to the receiver. The energy is used to supply a low power microcontroller for processing the message from the data concentrator and performing a measurement based on the attached sensor. Modulating the amplitude of the carrier signal is used for communication from data concentrator to wireless sensor. For reverse communication (WS to DC), the microcontroller modulates the load attached to the RX piezo. By monitoring the output power of the TX piezo amplifier, the information sent by the sensor module can be extracted. It has been demonstrated that the system is capable of transmitting up to 30 mW of power through a 7 mm thick aluminum barrier to the isolated sensor electronics, and half-duplex bidirectional data communication has been achieved at a data rate of 1 kbps. Further, a long-term performance and reliability assessment of this system were presented in [66] by performing an accelerated life testing. An estimate of the system lifetime operating at room temperature is about 90,000 h according to the extrapolation of experimental results.

Almost at the same time the research group at Rensselaer Polytechnic Institute extended the focus from ultrasonic communications through metal barriers to the development of a high performance system for through metal transmission of power and data [67,68,69,70,71,72,73,74,75,76,77,78,79,80,81,82,83]. This group has carried out in-depth research on this topic including modeling of this acoustic-electric channel, high-data-rate communication, and simultaneous power delivery and duplex communication, and *etc*. Their work has greatly propelled the researches of through-metal-wall power delivery and data transmission.

In [67], Shoudy *et al.* demonstrated an enhanced prototype of the “reflected-power” system for through-metal-wall power delivery and data transmission using two ultrasonic transducers resonant at 1 MHz and CW signal transmission and reflection. The system is similar to the one presented in [3]. There is also a MOSFET switch circuit has been designed to modulate the impedance inside the metal enclosure for data transmission from the inside to the outside. To accomplish power harvesting, power harvesting electronics are added to the inside isolated electronics circuit. The output electrical signal of the receiving transducer is first rectified and then stored as charge on a storage capacitor. The voltage on the capacitor is then regulated to the required voltage to operate the inside circuit. The control circuit monitors the voltage on the storage capacitor and enable or disenable the regulating circuit. Experimental results have shown that the power harvesting circuit is able to harvest 250 mW of power from the CW signal to power the sensors and electronics, and reliable communication at rates of up to 55 kbps is achievable through a 5.7 cm thick steel test block, which greatly exceeds the performance of the system presented in [3].

The power delivery and data transmission through the acoustic-electric channel similar to the diagram shown in Figure 5 has been studied in [68,69,70,71,72,73]. Finite element modeling and simulations of this system were performed in [68,69], A coupled stress strain and piezoelectric analysis were performed using an axis symmetric geometry to characterize the impedance transducer and system. The results showed an excellent correlation between the model and measured system [68]. The impacts of many design factors on the power transfer efficiency of the ultrasonic channel, including: transducer-wall coupling effects, transducer and wall resonance modes, transducer dimensions, and barrier composition and dimensions were simulated and strong correlation between the finite element simulations and the systems were also obtained [69]. All these simulation results have laid a baseline to properly construct such through-metal-wall power delivery and data transmission system. Analytical modeling of a sandwiched plate PT based acoustic-electric channel using the mixed domain two-port ABCD parameters were presented by Lawry *et al.* [70]. The developed model was proved to the ability of performing accurate channel performance prediction while reducing the computational complexity associated with finite element method.

In order to optimize the power transfer efficiency through the sandwiched metal plate PT channel, based on the linear two-port network model described using scattering parameters (S-parameters), simultaneous conjugate impedance matching was enforced on both ports of the channel to minimize electrical port reflections, and to maximize power transmission [71]. The experimental results have shown that maximum power transfer efficiency of 55% is achievable, with 81 W of power being transmitted linearly through one of the optimized channels.

In [72] a high temperature “reflected-power” ultrasonic through-metal-wall power delivery and data communication system was developed using a special high-temperature piezoelectric material, silicon-on-insulator electronics and a special housing package in order to maintain continuous operation while operating in a 260 °C environment. Measurements of the system have revealed that as much as 1 W of power can be transmitted through the steel wall and sensor data can be transmitted at a data rate of 50 kbps from the inside board to the outside at temperatures as high as 260 °C, which indicates that it is feasible for the proposed system to work in a high-temperature environment.

Differing from [45,46], where high power levels through acoustically thin (equal or less than the wavelength of the ultrasonic wave λ) metal wall was studied, Wilt *et al.* [73] performed experiments on acoustical high-power transmission through thick (>λ) metal walls using 25.4 mm diameter PZTs. The testing metal piece is stainless steel cylinder, with a diameter of 152.4 mm and 57.2 mm thickness. Experimental results have shown that the system is capable of transmitting large amounts of power (over 100 W) during long-term operation without failure. Extrapolation of the results indicates that it is highly feasible to transfer 1 kW of power through thick metal walls with larger diameter transducers.

In [74,75,76,77,78] this group developed novel systems capable of simultaneous high-power and high data-rate transmission through solid metal barriers using ultrasound. In these systems independent data and power channels were formed by two pairs of piezoelectric transducers only separated by 25.4 mm. Two transducers with 66.7 mm diameters and identical thickness resonances at 1 MHz were selected for the power channel, and the data communication channel was formed using two 25.4 mm diameter piezoelectric disc transducers with 4 MHz thickness mode resonances. A synchronous OFDM multi-carrier communication scheme was used to achieve a very high spectral efficiency and to ensure that there is only minor interference between the power and data channels. The full system is capable of transmitting data at a highest data rate of 17.37 Mbps and delivering 50 W of power through a 63.5 mm thick steel wall.

In [79] the work was extended to take account of the case where the channel consists of a steel-water-steel type of multilayer *i.e.*, a layer of water sandwiched between two steel walls. The channel was constructed by epoxy coupling 25.4 mm diameter circular piezoelectric transducers to one side of each of the two steel walls, spacing the steel plates a distance apart, and filling the intermediate region with water. The system was analyzed using a two-port model based on measured S-parameters and tested experimentally. Results indicate that a channel formed by two steel walls of 15.97 mm and 10.92 mm thickness separated by 88.3 mm water column is capable of transmitting data at a rate of 4 Mbps and of transferring power at an efficiency of over 30%.

In order to improve the operation feasibility and convenience in practical applications, a microcontroller-based handheld acoustic communication and power delivery through metallic barrier system was developed [80]. Successful data communication at 10 kbps between microcontrollers separated by a metallic wall was achieved, where the inside microcontroller was powered completely by the acoustic signal applied through the barrier.

Different from a pair of coaxially aligned PZTs configuration for the acoustic-electric channel, the power delivery and data communication channel was established by attaching a set of three ultrasonic transducers to the wall in [83]. An equivalent circuit model was presented based on PSpice^®^ simulation software, and the system’s power transfer and data transmission performance were simulated. The experiment results have shown a good agreement with the simulation results.

Besides the means of using ultrasonic piezoelectric transducers to form the acoustic-electric channel, a group at Newcastle University utilized electromagnetic acoustic transducers (EMATs) to generate ultrasonic waves via magnetostriction and the Lorenz force at the metallic barrier’s surface [5,6,84]. They achieved a transfer data rate of 1 Mbps across 25.4 mm thick metal wall with a transducer liftoff of 0.8 mm to the metal surface. This approach has the advantage of not requiring direct contact with the metal wall using couplant or permanent attachment to the metal wall. However, EMATs can be bulky compared to piezoelectric transducers and very inefficient. It is not practical to transfer useful available power through a metal wall using EMATs due to its extremely low efficiency of about 0.002%, so the authors used IPT instead for power delivery while using EMAT only for data communication with sensors [6].

## 4. Comparison of Various Ultrasonic Through-Metal-Wall Power Delivery and Data Transmission Systems

Up to now a wide variety of ultrasonic based through-metal-wall power delivery and/or data transmission systems have been developed. There are different modeling methods for the acoustic-electric channel and different configurations utilized in these systems. We provide a brief comparison and summary of these systems in the following subsections.

### 4.1. Methods for Modeling the Acoustic-Electric Channel

The goal in optimization of the performance of an ultrasonic through-metal-wall power delivery and data transmission system is to maximize the power transfer efficiency while achieving a high rate of accurate data transmission through the wall. There are many design factors influencing the system’s performance. The optimization process depends on the mathematical model of the whole system that captures the system’s dominant dynamic behaviors, so it is important to build an accurate model of the system for simulation of its performance during the design process of a system.

The acoustic-electric channel formed by a pair of piezoelectric transducers coaxially aligned opposite of the metal wall is the most common through-metal-wall power delivery and data transmission configuration as shown in Figure 5. There have been several different methods for modeling this channel and analyzing the voltage transfer ratio and power delivery efficiency, including analytical methods based on wave propagation equation and piezoelectric theory [34,35,36,37,38,39,40,41], finite element modeling [43,44,46,68,69], equivalent circuit method [2,42,43,44,45,46,47,48,49,50,51,83], S-parameters and ABCD parameters description of a linear two-port network model [70,71,75].

We have commented that the equivalent circuit method is superior to analytical methods based on wave propagation equations and linear piezoelectric theory because additional acoustic media such as coupling layers, insulation layers and electric circuits (power harvesting circuit, sensor conditioning circuit) can all be described using equivalent circuit model, while analytical methods will need a great deal of complex and difficult calculations which make it impractical to use them for the study of ultrasonic systems with a larger number of components.

Finite element model simulation which is a kind of theoretical analysis method is introduced to investigate the influence of various system design parameters on the system performance. Though the temperature analysis, acoustic field distribution in the metal wall and power transfer efficiency characteristic can all be obtained using finite element modeling with professional software like ANSYS^®^ and COMSOL^®^ Mutiphysics, there is a lack of understanding of the internal mechanism of the acoustic-electric channel.

The two-port network model of the channel described by using S-parameters or ABCD parameters can give similar results as the use of an equivalent circuit method. They are essentially the same in mapping the acoustic-electric channel to an equivalent electrical network. Electrically characterizing such a system can be very useful because a number of different transmitter and receiver electronic configurations can be designed and tested quickly and easily. Some critical performance metrics and characteristics of the channel can also be obtained. These methods slso require much less calculation than using FEM methods. The ABCD parameter modeling approach is especially useful when there are many layers forming the acoustic-electric channel because the whole channel model can be obtained just by multiplying the ABCD parameters matrix of each layer. Furthermore, the ABCD parameters model has also been used to predict the communication capability of the acoustic-electric channel [75]. Before the construction of a practical system, the equivalent circuit method, FEM methods, and ABCD parameters model are useful for performance prediction of the system. After the system has already been developed, the S-parameter modeling approach is convenient for calculation of the voltage transfer ratio and power transfer efficiency of the system.

### 4.2. Different Transducer Configuration and Communication Mode with Enclosed Sensors

**Figure 7:**
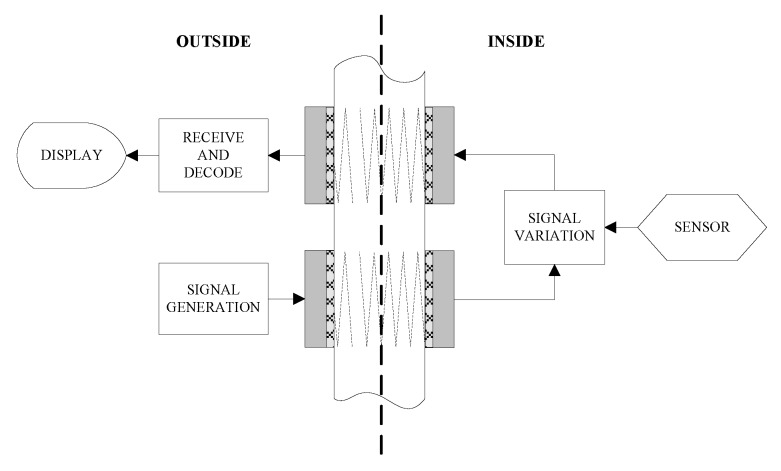
Diagram of two pairs of PZTs configuration, redrawn from Murphy [55].

An ultrasonic through-metal-wall power delivery and data transmission system should be able to get data from the sensors (inside to outside data transmission) and send data into inside sensors (outside to inside data transmission) with power transfer through the metal wall. There are different transducer configurations and communication mode to achieve bidirectional data transmission with the sensor. One of the configurations is to use two pairs of piezoelectric transducers to form two separated data transmission channels [55,56,77] as shown in Figure 7.

**Figure 8:**
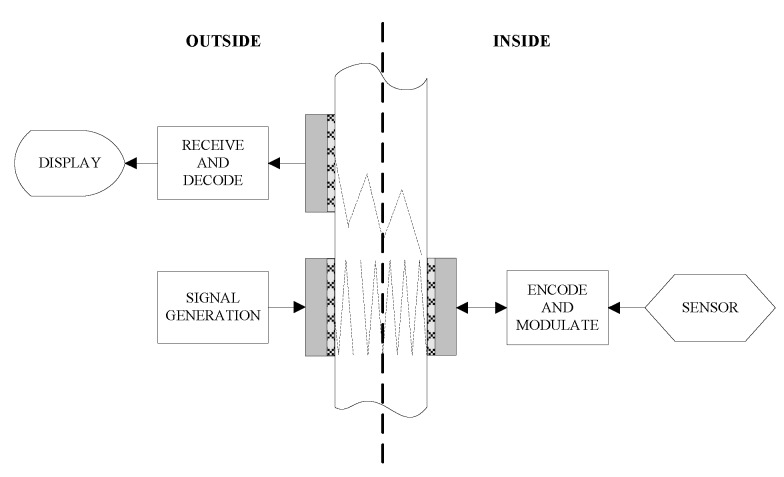
Diagram of three-transducer configuration, redrawn from Murphy [55].

In this configuration, the outside generated carrier signal is transmitted through the metal wall to the inside unit using one pair of transducers oppositely attached to the wall. Usually the amplitude modulation communication scheme is adopted here. The power can also be transmitted through the metal wall simultaneously over this outside-to-inside channel. The inside sensor reading is modulated and retransmitted to the outside unit using another pair of transducers. Hence, signals can be simultaneously transmitted through the metal wall in full-duplex mode. This configuration needs two transducers installed inside, so it is inconvenient when there is strict space constraint for the inside unit. The second is a three-transducer configuration [55,56,83], in which two piezoelectric transducers are used on the outside and a single transducers is used on the inside as shown in Figure 8.

The mode of data transmission from the outside to inside is the same as that in a two pairs of transducers configuration. The data transmission from the inside sensor to the outside unit is similar to the acoustic impedance modulation mode used in [3,67]. The acoustic wave injected into the metal wall from the outside transmitting transducer travels across the wall. As the acoustic wave reaches the interface of the inside wall and the inside transducer, a fraction of its energy is reflected and the remainder is absorbed by the inside transducer. The ratio of reflected to absorbed energy depends on the acoustic impedance match of the transducer to the wall. The reflected wave travels across the metal wall towards outside face of the wall, where it can be detected on the outside by the third transducer with decreased amplitude due to beam spread effects. The amplitude of the reflected wave can be modulated by changing the electrical load on the inside transducer that induces a variation of the acoustic impedance of the inside transducer. Thus the sensor reading carried in this reflected amplitude modulation wave can be recovered on the outside. Usually the bidirectional data transmission is performed in half-duplex mode meaning that at the same time there is only one-way data transmission.

Three-transducer configurations are still bulky and inconvenient. The most adopted configuration is to use a single pair of transducers for two-way data transmissions and power delivery from the outside to the inside. In this configuration, these two piezoelectric transducers are coaxially attached to the opposite sides of the metal wall. The principle for two-way data transmission is similar to the other two configurations. It is deemed that there is no need for a third transducer on the outside. The reflected amplitude modulated acoustic wave interacts directly with the outside transducer, modifying the electrical input impedance seen by the output of the power amplifier and producing envelope variations in the power amplifier output signal, so just by using the outside transmitting transducer to receive the reflected amplitude modulated acoustic wave and the sensor data from inside can be recovered. The majority of the publications only achieve half-duplex data transmission over this channel. The group at Rensselaer Polytechnic Institute proposed a novel way to achieve full-duplex data transmission [81,82] using a pair of coaxially aligned 25.4 mm PZTs with 1 MHz nominal resonant frequencies. Due to the channel frequency selectivity, a carrier frequency selection and tracking algorithm was presented to choose a frequency of operation at which both adequate power delivery and reliable full-duplex communication were achieved. A protocol for full-duplex communication through metal walls was also discussed. The outside-to-inside amplitude modulations and the outside-to-inside amplitude modulations can occur simultaneously on the same carrier. The system can allow for the continuous operation of internal electronics requiring consumption power of less than 100 mW. Sensor data can be transmitted at a rate in excess of 30 kbps.

### 4.3. Summary of Ultrasonic Through-Metal-Wall Power Delivery and Data Transmission Systems

A number of ultrasonic through-metal-wall power delivery and data transmission systems have been developed. It is necessary to give a brief summary of these studies. Table 1 summarizes these systems with the type of configuration, maximum power transfer efficiency, metal wall material and thickness, achievable maximum data transmission capability, and communication mode. 

**Table 1:** Summary of ultrasonic through-metal-wall power delivery and data transmission systems.

Systems	Configuration	Metal Wall and Thickness (mm)	Power Delivery		Data Rate (bps)	Data Transmission Mode
			Power Efficiency			
Sherrit *et al.* [42]	One pair of PZTs	Titanium (2.5 mm)	—	53%	—	—
Bao *et al.* [44]	One pair of 38 mm diameter PZTs	Titanium (3.4 mm)	100 W	88%	—	—
Bao *et al.* [45,46]	One pair PZT stacks (four 50.8 mm diameter PZTs in each stack)	Titanium (5 mm)	1068 W	80%–90%	—	—
Moss *et al.* [47,48]	One pair of 38 mm diameter PZTs	Aluminum (1.6–5 mm)	300 mW	30%	—	—
Moss *et al.* [49,50,51]	One pair of 38 mm diameter PZTs	Aluminum (1.6 mm)	420 mW	42%	115 k	Half-duplex
Murphy [55]	One pair of PZTs with power reflected mode	Steel (148 mm)	—	—	300	Half-duplex
Murphy [55]	Two pairs of PZTs	Steel (148 mm)	—	—	5 k	Full-duplex
Murphy [56]	Three PZTs	Steel (152.4 mm)	—	—	500	Half-duplex
Kluge *et al.* [3,65]	One pair of PZTs	Aluminum (7 mm)	30 mW	—	1 k	Half-duplex
Shoudy *et al.* [67]	One pair of 25.4 mm diameter PZTs	Steel (57 mm)	250 mW	—	55 k	Half-duplex
Lawry *et al.* [71]	One pair of PZTs with diameter from 12.7 mm to 66.7 mm	Steel (9.5–63.5 mm)	81 W	55%	—	—
Lawry *et al.* [72]	One pair of high temperature PZTs, operating at 260 °C	Steel (≈16 mm)	1 W	≈63%	50 k	Half-duplex
Wilt *et al.* [73]	One pair of 25.4 mm diameter PZTs	Stainless steel (57.2 mm)	>100 W	>60%	—	—
Lawry *et al.* [74,75,76,77,78]	Two pairs of PZTs	Steel (63.5 mm)	50 W	—	17.37 M	Full-duplex
Chakraborty *et al.* [79]	One pair of 25.4 mm diameter PZTs	Steel (15.97 mm)-water (88.3 mm)-steel (10.92 mm)	—	30%	4 M	Half-duplex
Ashdown *et al.* [81,82]	One pair of 25.4 mm PZTs	Steel (57.15 mm)	≈100 mW	—	>30 k	Full-duplex
Graham *et al.* [5,6,84]	One pair of EMATs with liftoff of 0.8 mm to the surface of the metal wall	Steel (25.4 mm)	—	—	1 M	Half-duplex

From the table it is clear that ultrasonic through-metal-wall power delivery and/or data transmission systems based on piezoelectric transducers represent the majority of studied systems, as there is only one system based on EMAT ultrasound. The main merits of PZT-based methods are their capability to achieve high power transfer efficiency and transmission data rates. While EMAT ultrasoound can not achieve available power transfer through metal barriers. The maximum amount of power delivery is over 1000 W, which is implemented in [45,46] through a 5 mm thick titanium barrier using a pair of PZT stacks. However it may be not suitable for powering enclosed embedded sensors because of its large volume. The system with the best performance is developed by Lawry *et al.* [74,75,76,77,78], and is capable of simultaneously transmitting power of 50 W and data at a rate as high as 17.37 Mbps using an OFDM digital communication scheme. However this system uses two pairs of piezoelectric transducers to form the separate power delivery and data transmission channel. The best performance for through non-ferromagnetic metal wall power and data transmission using a pair of piezoelectric transducers is achieved in [50], which reports the simultaneous transmission of power at a level of 420 mW and data at rates as high as 115 kbps through 1.6 mm thick aluminum plate. The best performance for through ferromagnetic metal walls is simultaneous transmission power of 250 mW and data at a rate of 50 kbps through a 57 mm thick steel wall [67]. The system allowing for full-duplex data transmission with the sensor and power delivery through metal wall using a pair of PZTs was only achieved by the group at Rensselaer Polytechnic Institute [81,82]. Nevertheless the system is very frequency sensitive and needs a complex frequency tracking algorithm.

## 5. Concluding Remarks

Powering and communication with sensors that are embedded in sealed conductive materials without penetration is essential in industrial, aeronautic and aerospace applications. This review has introduced a number of alternative systems that have been demonstrated to be able to fulfill through-metal-wall power delivery and/or data transmission with sensors enclosed in hermitical containers.

### 5.1. Characteristics of Through-Metal-Wall Power Delivery and Data Transmission Systems

Among these previous systems, one category of systems, which includes inductive coupling, capacitive coupling, and magnetic resonance coupling approaches, is based on the electromagnetic coupling principle.

IPT and inductive data transmission through metal walls have been demonstrated in several systems. Due to the influence of the Faraday shielding effect the inductive coupling approach is applicable to applications with low conductivity, low permeability materials such as thin aluminum or stainless steel walls. As the skin depth of the material increases rapidly with the increase of frequency, these systems usually operate at relative low frequencies such as several kilohertz. Another advantage of the inductive coupling approach is that the system can operate at temperatures far exceeding the Curie point of a piezoelectric material [6], at which piezoelectric transducers would lose their functionality. The CPT/CPPT approach achieves power delivery based on electric field coupling, but the capacitive coupling structure formed by two pairs of coupling plates is bulky. Though there is system demonstrating wireless power transfer through metal barriers to multiple loads based on CPT/CCPT, the metal barriers between each of the coupling plates should be separate. This point limits the practicality of this approach in engineering applications. Furthermore, there is no data transmission information using CCPT up to now. Through-metal-wall power delivery using the magnetic resonance coupling approach is rarely studied, but it has been reported that 3 W of power can be transferred through a 1 mm thick stainless steel wall at an efficiency of approximately 40% over a distance of 12 cm. However there are also no literature reports about through-metal-wall data transmission using magnetic resonance coupling. The biggest advantage of electromagnetic coupling methods is that there is no need for the transmitting and receiving unit to contact directly with the metal wall. The surface of the metal wall never has to be processed before performing power and/or data transmission. While the common limitation to electromagnetic coupling is that for high permeability metal such as carbon steel wall, it is hard to perform effective power delivery through the barrier.

Besides electromagnetic coupling-based methods, the ultrasound-based methods are the major means used for through-metal-wall power delivery and data transmission due to the good characteristic of ultrasonic wave propagating in a wide variety of metal materials. There are two types of ultrasonic systems, PZT-based systems and EMAT-based systems. PZT-based systems are by far the most widely used for through-metal-wall power and data transmission due to the high power delivery efficiencies they can provide and the possibility of miniaturizing their designs. PZT-based systems can be used for power delivery alone, for data transmission alone, as well as for simultaneous power delivery and data transmission through metal walls. Another advantage of PZT-based approaches is that there are no electromagnetic interference problems since just acoustic waves are used. The main limitation of PZT-based system is that they require direct coupling of piezoelectric transducers with metal walls to provide a good acoustic transmission path and system performance is highly reliant on the quality of the coupling. Poor coupling will introduce severe impedance mismatch over the acoustic-electric channel and cause the power transfer efficiency and transmission data rate to decrease rapidly. EMAT is a non-contact approach and provides an effective solution for robust through-metal-wall data transmission. However, the power transfer efficiency for EMAT is too low, which makes it unsuitable for power delivery through metal walls. On the other hand, EMAT can also operate at high temperatures far exceeding that of piezoelectric transducers [6].

**Figure 9:**
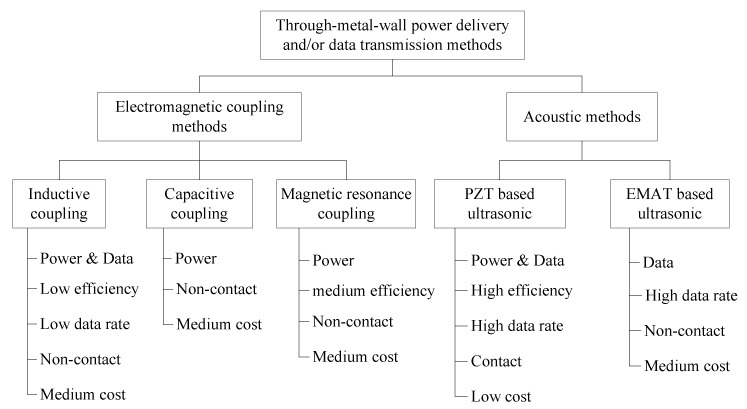
Summary of characteristics of different through-metal-wall power delivery and/or data transmission methods.

To make a clear comparison, a summary of characteristics of different through-metal-wall power delivery and data transmission methods reviewed are illustrated in Figure 9. The tree-style taxonomy describes each technique from the point of view of various aspects including the coupling type, the power and data transfer ability, the power delivery efficiency, the achievable data rate, the contact with the metal barrier requirement and the cost.

### 5.2. Challenges and Developing Trends of Through-Metal-Wall Power Delivery and Data Transmission Technology

The electromagnetic coupling methods are suitable for applications where the barrier is just a thin non-ferromagnetic metal wall and there is enough space for installation of the coils or capacitive plates. In order to miniaturize the design, further investigations on the design of small size coils, which can be used without a dramatic reduction of the power transfer efficiency, is required. Meanwhile new techniques based on electromagnetic coupling are still coming forth. For example, the sheet-like waveguide-based system utilizes the concept of evanescent waves for power and data transmission through metal walls [85]. The transmission sheet operates at a resonance frequency as high as 25 MHz and it can achieve a higher data rate than the magnetic resonance coupling method.

Generally speaking, ultrasound-based systems are superior to electromagnetic coupling-based systems because ultrasonic waves can propagate not only through non-ferromagnetic metal but also through ferromagnetic metal walls. Furthermore higher power transfer efficiency and higher transmission data rates can be achieved based on ultrasonic systems. However, even for ultrasound-based systems, the majority are experimental demonstration systems while actual practical applications are fairly rare. We have mentioned previously that the PZT-based technique has been adopted in NASA’s Mars Sample Return Mission, but the plan is underway, and there is no more information about the evaluation of the technique. There are still several problems that need to be solved to improve practicality in engineering applications.

The first problem for PZT-based ultrasonic systems is the coupling quality for long term reliability. It is known that the major drawback of piezoelectric transducers is the need for coupling between transducers and metal barrier surface. Since the metal wall tends to be corrosive over the long term and the coupling layers such as epoxy may degrade or fail as operating time goes by., all these factors will result in severe impedance mismatch of the channel. Therefore, alternative couplants with stable long term properties are a key point and should be investigated in further research. The second is high temperature operation conditions. As we know, the maximum working temperature of a piezoelectric transducer is determined by the Curie point, at which the piezoelectric material loses its function. The Curie point typically varies within a range from 210 °C to 365 °C [6]. In consistently high temperature operating environments such as nuclear and chemical process applications, we should chose piezoelectric material with a high Curie point and make the transducer operate at a temperature far below that Curie point. The third is self-adaptation to the characteristic variation of the acoustic-electric channel. The power transfer efficiency through the metal is highly frequency dependent. The temperature fluctuations in some applications will affect the speed of ultrasonic waves in the metal and cause a change of the acoustic characteristics of the channel. This finally will result in a deviation of the optimal operating frequency for the piezoelectric transducer. As shown in [67], a very small change of the operating frequency from 1.087 MHz to 1.021 MHz will cause a 10-fold power transfer efficiency decrease. Therefore the optimal frequency tracking algorithm need to be further studied for reliable long-term operation.

Another trend is the combination of two or more methods to fulfill through-metal-wall power and data transmission. In [6] an ultrasonic EMAT and IPT combination has been used for data transmission and power delivery through metal, respectively. When there is no strict volume restriction, we believe that such hybrid systems using combinations of more than a single technique can provide more reliability in practical engineering applications. We conclude that a number of systems have been demonstrated to be able to power and communicate with isolated sensors through metal barriers. With the development of ongoing research, more mature, practical and reliable systems will be used to achieve through-metal-wall power delivery and data transmission with sensors enclosed in sealed containers in the near future.
